# Evaluation of Dose-Intense Ifosfamide, with and Without Edatrexate, in Adults with Sarcoma

**DOI:** 10.1080/13577149977758

**Published:** 1999-09

**Authors:** Ephraim S. Casper, Gary M. Schwartz, Denis Leung, Alison Sugarman, Joseph R. Bertino

**Affiliations:** 1 Division of Solid Tumor Oncology Department of Medicine Memorial Sloan-Kettering Cancer Center NewYork NY 10021 USA; 2 The Department of Biostatistics and Epidemiology Memorial Sloan-Kettering Cancer Center NewYork NY 10021 USA; 3 Medical Oncology Memorial Sloan-Kettering Cancer Center at St. Clare's 23 Pocono Road Denville NJ 07834 USA

## Abstract

*Purpose.* To define the maximally tolerated dose (MTD) of ifosfamide when given with G-CSF on an every other week schedule, and to define the MTD of edatrexate that can be given every two weeks with an intense schedule of ifosfamide.

*Patients and Methods.* Forty-one patients with metastatic or unresectable, locally advanced sarcoma participated in this 2-step phase I trial.The starting dose of ifosfamide was 10 gm/m^2^ given by continuous intravenous infusion over 4 days every 2 weeks.When the MTD was defined, edatrexate, beginning at a dose of 40 mg/m^2^ intravenously every 2 weeks was added in subsequent cohorts of patients.

*Results.* Myelosuppression was the most prominent toxicity. Fatigue, nausea, and vomiting were observed in the majority of patients. Ifosfamide 12 gm/m^2^ given every 2 weeks approached or exceeded the MTD. Edatrexate 100 mg/m^2^ could be given safety as an intravenous bolus with ifosfamide 10 gm/m^2^ every 2 weeks. Therapeutic responses were observed in patients with measurable disease.

*Conclusions.* This study demonstrates the feasibility of administering a dose-intense schedule of ifosfamide alone or ifosfamide with edatrexate that might be applied in the adjuvant or neo-adjuvant setting.


Sarcoma (1999) 3, 121–127

ORIGINAL ARTICLE

### Evaluation of dose-intense ifosfamide, with and without edatrexate, in adults with sarcoma

EPHRAIM S. CASPER[1], GARY M. SCHWARTZ[1], DENIS LEUNG[2], ALISON SUGARMAN[1] & JOSEPH R. BERTINO[1]

*Division of Solid Tumor Oncology, Department of Medicine[1], and the Department of Biostatistics and Epidemiology[2], Memorial Sloan-Kettering Cancer Center, New York, NY 10021, USA*

**Abstract**

*Purpose.* To define the maximally tolerated dose (MTD) of ifosfamide when given with G-CSF on an every other week schedule, and to define the MTD of edatrexate that can be given every two weeks with an intense schedule of ifosfamide.

*Patients and Methods.* Forty-one patients with metastatic or unresectable, locally advanced sarcoma participated in this 2-step phase I trial. The starting dose of ifosfamide was 10 gm/m$^2$ given by continuous intravenous infusion over 4 days every 2 weeks. When the MTD was defined, edatrexate, beginning at a dose of 40 mg/m$^2$ intravenously every 2 weeks was added in subsequent cohorts of patients.

*Results.* Myelosuppression was the most prominent toxicity. Fatigue, nausea, and vomiting were observed in the majority of patients. Ifosfamide 12 gm/m$^2$ given every 2 weeks approached or exceeded the MTD. Edatrexate 100 mg/m$^2$ could be given safety as an intravenous bolus with ifosfamide 10 gm/m$^2$ every 2 weeks. Therapeutic responses were observed in patients with measurable disease.

*Conclusions.* This study demonstrates the feasibility of administering a dose-intense schedule of ifosfamide alone or ifosfamide with edatrexate that might be applied in the adjuvant or neo-adjuvant setting.

### Introduction

The activity of ifosfamide in soft tissue sarcoma has been recognized since 1973, but the development of this drug was hindered by its urothelial toxicity. The introduction of the disulfide uroprotective agent, mesna, permitted extensive clinical evaluation of ifosfamide. In a large phase II trial, the response to ifosfamide 8 gm/m$^2$ was 21% among 124 patients with sarcoma.[1] In a randomized phase II trial, the response to ifosfamide 5 gm/m$^2$ was 24% among patients with soft tissue sarcoma who had not previously been treated with chemotherapy.[2] Response to its analog, cyclophosphamide, at a dose of 1.5 gm/m$^2$ was only 9%, in spite of greater myelosuppression in the latter group of patients.

A dose–response relationship has been claimed for ifosfamide in patients with soft tissue sarcoma.[3] In most studies of high dose ifosfamide, the drug has been given at increased doses every 3 to 4 weeks. Colony stimulating factors, such as G-CSF[3] and GM-CSF,[4,5] reduce the myelosuppressive toxicity of high dose ifosfamide, but dose-dependent renal and neurologic toxicities limit the dose of ifosfamide that can be given per cycle.[6] Most investigators have attempted to increase dose-intensity by increasing the total dose of ifosfamide given over several days every 3 weeks. In this study, we explored intensification of ifosfamide therapy by increasing the frequency of ifosfamide administration to every 2 weeks.

As part of our phase II program for patients with soft tissue sarcoma, we observed 5 major objective responses to edatrexate among 35 patients with soft tissue sarcoma.[7] The starting dose of edatrexate in that trial was 80 mg/m$^2$ weekly, but patient tolerance was variable, and weekly doses ranged from 50 to 120 mg/m$^2$. Edatrexate also has shown activity in patients with sarcoma in an Eastern Cooperative Oncology Group phase II trial.[8] In addition, edatrexate has demonstrated antineoplastic activity in human sarcoma cell lines in our laboratory.[9] Preclinical data have suggested synergy between edatrexate and alkylating agents in several murine tumor models, including T241 sarcoma.[10] We reasoned that the combination of edatrexate and ifosfamide might have useful clinical efficacy in patients with sarcoma. Furthermore, an active, dose-intense ifosfamide-based combination might be a useful component of neoadjuvant or adjuvant chemotherapy

Correspondence to: Ephraim S. Casper, Chief, Medical Oncology, Memorial Sloan-Kettering Cancer Center at St. Clare's, 23 Pocono Road, Denville, NJ 07834, USA. Fax: 973 983-7330; E-mail: caspere@mskcc.org

1357-714X print/1369-1643 online/99/020121-07 © 1999 Taylor & Francis Ltd regimen for use in sequence or alternating with doxorubicin.

A two-stage phase I trial of high dose ifosfamide, therefore, was conducted. First, the maximum tolerated dose (MTD) of ifosfamide given by continuous intravenous infusion over 4 days every other week with G-CSF support was determined. We then sought the MTD of edatrexate that could be given safely every two weeks with an intense schedule of ifosfamide. We now report the results of this investigation.

### Patients, materials and methods

The protocol was reviewed and approved by the Institutional Review Board of Memorial Sloan-Kettering Cancer Center.

### *Patients*

This study was designed as a disease-specific phase I trial for patients with metastatic or unresectable, locally advanced sarcoma. Histologic confirmation of sarcoma by the Department of Pathology of Memorial Hospital was necessary. The trial was limited to patients who had not previously been treated with ifosfamide, and those with not more than one previous chemotherapy regimen. Patients could not have received chemotherapy or therapeutic irradiation during the 4 weeks before their first dose of ifosfamide. A Karnofsky performance status of 60% or greater was required, as was a WBC≥4000/mm$^3$, platelet count≥150,000/mm$^3$, serum bilirubin<1.5 mg/dl, and serum creatinine<1.5 or creatinine clearance≥60 mg/min/1.73m$^2$. Patients with clinically evident third space fluid (ascites or pleural effusions) were excluded from this trial.

All patients had a physical examination, a chest radiograph, and appropriate imaging studies to establish extent of disease and tumor measurements when possible. Measurable or evaluable disease was not required, however.

### *Therapeutic agents*

Ifosfamide and mesna were purchased as Ifex and Mesnex, respectively (Mead Johnson Oncology Products, Evansville, IN). Ifosfamide was reconstituted with Sterile Water for Injection USP to yield a 50 mg/ml solution. The daily prescribed dose of ifosfamide was mixed in 1 L of 0.45% NaCl, and was administered intravenously over a 24 hour period. A dose of mesna equal to the ifosfamide dose was added to the IV bag containing the ifosfamide.

The Division of Cancer Treatment (National Cancer Institute, Bethesda, MD) supplied edatrexate as a lyophilized sterile powder in 50 mg vials. Edatrexte was dissolved in 4 ml sterile Normal Saline USP to yield a solution of edatrexate 12.5 mg/ml. The final dose of edatrexate was diluted in a 50 ml bag of Normal Saline USP, and administered intravenously over 25–30 min.

Recombinant human filgrastim (G-CSF, Neupogen) for this study was provided by Amgen, Inc. (Thousand Oaks, CA) under an agreement with Memorial Sloan-Kettering Cancer Center.

### *Treatment plan*

Signed informed consent was obtained from all patients who participated in this trial. Treatment was given in the hospital. Oral or intravenous hydration was administered to maintain a urine output of at least 2000 ml/24 h during ifosfamide infusion. The ifosfamide and mesna were administered by continuous 24-hour intravenous infusion over 4 consecutive days every 2 weeks. Mesna was not continued beyond the completion of the ifosfamide infusion. Ifosfamide therapy was interrupted for hematuria (RBC greater than 11 RBC/hpf), an increase in serum creatinine>1.0 mg/dl, or for clinically significant neurological side effects. The initial dose of ifosfamide was 10 gm/m$^2$. In patients who received edatrexate, this agent was given on the first day of each cycle. The initial dose of edatrexate was 40 mg/m$^2$.

G-CSF (5μ/kg/day) was self-administered subcutaneously daily beginning 48 hours after the completion of the ifosfamide infusion, and was administered until the recovering ANC was ≥1500/mm$^3$. Subsequent doses of ifosfamide were not administered within 24 hours of G-CSF injection. The 4-day course of therapy followed by G-CSF was defined as a cycle of therapy.

If on day 15 the WBC was <4000/mm$^3$ the ANC was <1500/mm$^3$ or the platelet count was <150,000/mm$^3$, or if grade 2 or greater mucositis was present, chemotherapy was held one week. If toxicity had not resolved by day 21, treatment was held one additional week. Patients were taken off study if hematologic recovery was delayed beyond this time. At least 3 patients were to be treated at each dose level. Dose escalation within individual patients was not permitted. If dose-limiting toxicity was seen in the initial 3 patients at a given dose level, additional patients were added to further evaluate the toxicity. In the original plan, the maximum tolerated dose (MTD) was defined on the basis of duration of myelosuppression, with more than 5 days of grade 4 neutropenia constituting dose-limiting toxicity. During the study it became clear that the non-hematologic toxicity likely to be associated with that degree of myelosuppression would be unacceptable. Thus, since our objective was to treat patients every 2 weeks, any toxicity that prevented recycling of treatment on day 15 (e.g. ANC<1500/mm$^3$ or platelet count<150,000/mm$^3$) was accepted as dose-limiting, and the MTD was defined as the highest dose level at which the majority of patients could be recycled on day 15.

This was a two-part study. First, the MTD and schedule for the administration of ifosfamide every 2 weeks was defined. Additional cohorts of patients were then treated with escalating doses of edatrexate in combination with ifosfamide to determine the MTD of edatrexate for use in combination. One dose below the MTD of ifosfamide defined in the first part of the study was to have been used as the constant in determining the MTD for edatrexate in the second part of the study.

### *Evaluation during study*

Patients were followed until dose-limiting toxicity precluded further therapy, or until progression of disease was observed. An automated CBC and platelet count was performed twice weekly. Physical examination and assessment of toxicity was done before each cycle of ifosfamide. A biochemical profile including electrolytes, BUN, creatinine, calcium, phosphorus, and hepatic enzymes was obtained every 2 weeks. Measurement of indicator lesion(s) was performed at least every eight weeks for patients with measurable disease.

### *Criteria for therapeutic response and toxicity*

The NCI Common Toxicity Criteria were used in this trial. Therapeutic response was not the principal endpoint of this trial. However, for those patients who had measurable disease, the criteria of the Adult Intergroup Soft-Tissue Sarcoma Committee were applied.[11] The duration of response was measured from the first day of therapy. Unequivocal clinical deterioration as evident from increasing pain, progressive weight loss and falling performance status was accepted as an indication of disease progression in the absence of significant change in measured lesions. In patients with evaluable disease, unequivocal tumor shrinkage was recorded as improvement.

### Results

The characteristics of the 41 patients who participated in the trial are presented in Table 1. The primary sites for the 34 patients with non-osseous sarcoma are listed in Table 2. All patients had a Karnofsky performance status of 70% or greater, and 78% had no previous chemotherapy. With the exception of one patient whose prior therapy was only paclitaxel, the previously treated patients had all been treated with doxorubicin or a doxorubicin-based combination. Two patients were inevaluable for hematologic toxicity. One had acute central nervous system toxicity during his infosfamide infusion, and therapy was discontinued. Another was ineligible because he was found to have a pleural effusion shortly after receiving his first doses of therapy. All but 3 evaluable patients had measurable disease. An additional patient was lost to follow-up before post-treatment tumor measurements could be made.

### *Toxicity*

Table 3 demonstrates the WBC and platelet toxicity of the first cycle of chemotherapy for each dose level. Table 4 outlines the WBC and platelet toxicity encountered when all cycles administered at each dose level are included.

The first two levels involved infosfamide without edatrexate. The starting dose of ifosfamide, 10 gm/m$^2$, was well-tolerated. At 12 gm/m$^2$, one episode of septicemia was encountered, and sufficient myelosuppression was observed in the other 2 patients that a decision was made to interrupt accrual of patients at that level. Based on the plan to begin escalation of edatrexate in combination with a dose of infosfamide one level below the MTD, the dose of infosfamide

**Table 1**. *Characteristics of 41 patients treated with ifosfamide + G-CSF =/– edatrexate*

| | | | | |
|---|---|---|---|---|
| **Age** | | **Histologic diagnosis** | | |
| Median | 50 years | Soft tissue sarcoma | | 34 |
| Range | 24–73 years | MFH* | 8 | |
| | | Leiomyosarcoma | 5 | |
| **Performance status (Karnofsky)** | | Synovial sarcoma | 5 | |
| | | MPNT* | 3 | |
| median | 80% | Liposarcoma | 2 | |
| range | 70–90% | Epitheliod sarcoma | 2 | |
| | | Fibrosarcoma | 2 | |
| **Sex** | | GIST* | 2 | |
| | | Hemangiopericytoma | 1 | |
| Male | 25 | Spindle Cell | 1 | |
| Female | 16 | Rhabdomyosarcoma | 1 | |
| | | Chondrosarcoma | 1 | |
| **Prior chemotherapy** | | Undifferentiated | 1 | |
| Chemotherapy | 9 | | | |
| (includes 5 with prior RT) | | Bone sarcoma | | 7 |
| No chemotherapy | 32 | Chondrosarcoma | 4 | |
| | | MFH* | 1 | |
| | | Osteogenic sarcoma | 1 | |
| | | Angiosarcoma | 1 | |

*MFH, malignant fibrous histiocytoma; MPNT, malignant peripheral nerve tumor; GIST, gastrointestinal stromal tumor.

**Table 2.** *Primary sites of soft tissue sarcoma in 34 patients treated with ifosfamide + G-CSF +/– edatrexate*

| | | | | | |
|---|---|---|---|---|---|
| **Extremity/superficial trunk** | | 20 | **Head/neck sarcoma** | | 2 |
| MFH* | 6 | | Leiomyosarcoma | 1 | |
| Synovial sarcoma | 5 | | MPNT | 1 | |
| Epithlioid | 2 | | **Retroperitoneal/pelvic** | | 5 |
| Fibrosarcoma | 1 | | MFH* | 2 | |
| Liposarcoma | 1 | | Liposarcoma | 1 | |
| MPNT* | 1 | | MPNT* | 1 | |
| Leiomyosarcoma | 1 | | GIST* | 1 | |
| Rhabdomyosarcoma | 1 | | **Genitourinary sarcoma** | | 2 |
| Chondrosarcoma | 1 | | Leiomyosarcoma | 1 | |
| Undifferentiated | 1 | | Spindle Cell | 1 | |
| | | | **Other** | | 1 |
| | | | Cardiac fibrosarcoma | 1 | |
| **Gastrointestinal sarcoma** | | 2 | **Unknown** | | 2 |
| Leiomyosarcoma | 1 | | Hemangiopericytoma | 1 | |
| GIST* | 1 | | Leiomyosarcoma | 1 | |

**Table 3.** *Response*

| | Soft tissue sarcoma | Bone sarcoma |
|---|---|---|
| Complete response | 0 | 1 |
| Partial response | 4 | 1 |
| Minor response | 2 | 0 |
| Stable disease | 14 | 4 |
| Improvement (evaluable disease) | 3 | 0 |
| Progression of disease | 9 | 0 |
| Not evaluable | 2 | 1 |
| Total | 34 | 7 |

selected for study in combination with edatrexate was 8 gm/m$^2$ every 2 weeks.

It is difficult to draw firm conclusions regarding the relationship between dose and hematologic toxicity given the wide range of WBC, ANC, platelet nadirs, and the limited range of ifosfamide doses studied. Overall, however, there seemed to be a threshold effect for edatrexate, with doses up to 80 mg/m$^2$ having little impact on myelosuppression. With edatrexate 100–120 mg/m$^2$, the addition of edatrexate to a given dose of ifosfamide appeared to be associated with greater myelotoxicity.

In addition, 15 patients had grade 3 or 4 anemia. Also anemia was not clearly related to dose level, although it did appear related to duration of therapy. All patients experienced alopecia. The incidence of the other common non-hematologic toxicities, nausea and vomiting, mucositis, and fatigue is shown in Table 5. Paralleling the anemia observed, fatigue appeared to be associated with prolonged duration of therapy.

In combination with ifosfamide 8 gm/m$^2$, it was possible to escalate the dose of edatrexate to 100 mg/m$^2$, the phase II dose of that agent for weekly administration. Therefore, it was elected to treat a cohort of patients with infosfamide 10 gm/m$^2$, while keeping the dose of edatrexate at 100 mg/m$^2$. When

**Table 4.** *Hematologic toxicity for all cycles of ifosfamide + G-CSF +/– edatrexate*

| Ifosfamide (g/m$^2$) | Edatrexate (mg/m$^3$) | Number of patients | Number of cycles | Median WBC nadir (range) | Median ANC nadir (range) | Median platelet nadir (range) | Median number of cycles per patient (range) |
|---|---|---|---|---|---|---|---|
| 10 | 0 | 5 | 23 | 0.8 (0.1–46.9) | 0.22 (0.0–42.8) | 173 (62–327) | 4 (3–6) |
| 12 | 0 | 3 | 7 | 1.0 (0.1–41.4) | 0.76 (0.0–35.19) | 149 (19–229) | 2 (1–4) |
| 8 | 40 | 5 | 22 | 6.6 (0.7–18) | 5.1 (0.26–16.92) | 162.5 (40–583) | 4 (2–8) |
| 8 | 60 | 7 | 28* | 3.6 (0.6–52.8) | 2.5 (0.2–46.46) | 145 (40–343) | 4 (1–7) |
| 8 | 80 | 6 | 30 | 1.75 (0.4–9.4) | 0.95 (0.05–7.9) | 183 (108–345) | 5 (1–7) |
| 8 | 100 | 5 | 17 | 3.0 (0.4–9.4) | 1.99 (0.05–7.9) | 158 (108–345) | 2 (1–7) |
| 10 | 100 | 5 | 22 | 2.0 (0.1–13.6) | 0.83 (0.0–9.79) | 95 (36–153) | 4 (4–6) |
| 10 | 120 | 5 | 19 | 1.8 (0.4–6.2) | 0.48 (0.01–4.85) | 196 (37–298) | 4 (1–7) |

*There are 27 evaluable cycles for this dose level.

**Table 5.** *Number of patients with selected non-hematologic toxicities for **all** cycles of ifosfamide + G-CSF +/– edatrexate*

| | | | # of | Mucositis | | Nausea/vomiting | | Fatigue | |
|---|---|---|---|---|---|---|---|---|---|
| Ifos | Edam | # of pts | cycles | Grade 2 | Grade 3 | Grade 2 | Grade 3 | Moderate | Severe |
| 10 | 0 | 6 | 24 | 0 | 0 | 1 | 0 | 4 | 0 |
| 12 | 0 | 3 | 7 | 0 | 0 | 1 | 2 | 2 | 1 |
| 8 | 40 | 5 | 22 | 2 | 0 | 2 | 0 | 2 | 0 |
| 8 | 60 | 7 | 28 | 2 | 0 | 4 | 0 | 2 | 0 |
| 8 | 80 | 6 | 30 | 2 | 1 | 1 | 0 | 2 | 0 |
| 8 | 100 | 5 | 17 | 0 | 2 | 1 | 0 | 3 | 1 |
| 10 | 100 | 5 | 22 | 2 | 0 | 3 | 0 | 0 | 5 |
| 10 | 120 | 5 | 19 | 4 | 0 | 2 | 1 | 3 | 2 |

tolerance of that combination was demonstrated, the dose of edatrexate was escalated to 120 mg/m$^2$. At that dose level, the majority of patients could not be recycled on day 15. Thus, it was concluded that ifosfamide 10 gm/m$^2$ with edatrexate 100 mg/m$^2$ every 2 weeks was a feasible schedule for further testing.

### *Therapeutic responses*

The endpoint of this trial was not therapeutic response, and phase II conclusions should not be drawn from a phase I trial. Nonetheless, the overwhelming majority of patients with soft tissue sarcoma had received on previous chemotherapy, and most were evaluable for therapeutic response (Table 6). It is impossible to evaluate duration of response, since the length of treatment on this protocol was generally brief, and therapy after completion of the protocol treatment was not uniform. Among the responders, however, one patient with an angiosarcoma of the pelvis is recorded as having experienced a clinical PR. He had no viable tumor at the time of resection after treatment with ifosfamide plus edatrexate. Although that patient had previously undergone resection of a metastatic lymph node in the axilla, he remains free of active sarcoma 48+ months after initiation of ifosfamide therapy. The response rate was 14% among the 29 patients with measurable soft tissue sarcoma. In addition, 3 patients with evaluable, but not strictly measurable disease experienced unequivocal antitumor responses. This 18% response rate observed among the 32 patients with evaluable soft tissue sarcoma, and the 24% response rate overall is similar to that reported for less intense schedules of ifosfamide alone.

**Table 6.** *Therapeutic response in 41 patients with sarcoma*

| | Soft tissue sarcoma | Bone sarcoma |
|---|---|---|
| Complete response | 0 | 1 |
| Partial response | 4 | 1 |
| Minor response | 2 | 0 |
| Stable disease | 14 | 4 |
| Improvement (evaluable disease) | 3 | 0 |
| Progression of disease | 9 | 0 |
| Not evaluable | 2 | 1 |
| Total | 34 | 7 |

### Discussion

The number of drugs with reproducible activity in patients with soft tissue sarcoma is limited, and the activity of the most active agents is modest at best. In large, randomized trials, even combination chemotherapy yields responses in fewer than 35% of patients.[12–14] In the absence of novel drugs with meaningful clinical activity, it is essential to maximize the therapeutic benefit of the available agents.

The antineoplastic activity of ifosfamide in patients with sarcoma is well-established. In virtually all studies of ifosfamide or ifosfamide based combinations, the drug has been given every 3–4 weeks. Phase II trials of ifosfamide 5–8 gm/m$^2$ yielded responses in 18–24% of patients with sarcoma,[1,2] and ifosfamide-based regimens have become a standard in the treatment of patients with sarcoma. Drawing phase III conclusions from phase I or phase II data is unreliable. Taken together, however, several lines of evidence from phase I and phase II trials suggest a dose–response relationship for ifosfamide in patients with sarcoma.

High response rates have been reported in trials of higher doses of ifosfamide. In a small cohort of patients with synovial cell sarcoma, a 100% CR+PR to infosfamide 14 g/m$^2$ was reported.[15] In a phase I trial in which the dose of ifosfamide was escalated from 8 to 18 g/m$^2$ in sequential cohorts of patients, the maximal tolerated dose was estimated to be 16 g/m$^2$.[6] Among 20 patients with sarcoma in that trial, 7 (35%) had major responses. More recently a 43% CR+PR rate was reported among 34 evaluable patients who received ifosfamide 14 gm/m$^2$ as first-line therapy.[16]

In a phase II trial, ifosfamide 14 g/m$^2$ given over three days by continuous infusion yielded responses in 29% of 37 patients with soft tissue sarcoma, and 40% of patients with bone sarcoma.[3] Also within that report was a small cohort of patients in which the same total dose of ifosfamide was given by intermittent bolus infusion. Five of 11 patients with soft tissue sarcoma as well as 3 of 3 patients with bone sarcoma responded, leading the authors to suggest that bolus therapy is more efficacious than continuous infusion.

Pharmacokinetic studies, however, have not shown a difference in area under the curve for serum ifosfamide or its metabolites, or in ifosfamide metabolites in urine for 1 hour or bolus infusions of ifosfamide.[27]

Further support for a dose–response relationship comes from studies in which responses to higher doses of ifosfamide yield responses after progression on lower doses. For example, ifosfamide 12 g/m$^2$ yielded responses in 33% of 36 patients who had progressed after prior doxorubicin and/or ifosfamide therapy with less than 8 g/m$^2$ per cycle.[18] It should be noted that not all patients in such studies have been shown to be truly refractory to the lower doses—not all clearly progressed under treatment.

Finally, high response rates have been seen in groups of patients treated with intensive anthracycline/ifosfamide regimens.[19,20] Although the relative contributions of the high doses of ifosfamide and the high doses anthracycline in the favorable results in such trials is unclear, the seeming lack of benefit for dose-escalation of doxorubicin in combination with modest doses of ifosfamide.[21] suggests that it is the intensity of ifosfamide therapy that is responsible.

Studies of high-dose therapy are likely to be biased toward younger patients of good performance status. Furthermore, many investigators have observed that gastrointestinal leiomyosarcoma is refractory to ifosfamide-based regimens, and that synovial sarcoma tends to be particularly responsive to such treatments. Leiomyosarcoma accounts for 35–50% of patients in many large phase II or phase III trials.[12,13] Thus, selection of younger patients with extremity sarcoma for phase I and pilot phase II studies may be responsible, at least in part, for the differences observed in response rate among various studies.

A conclusion regarding the relative efficacy of high-dose ifosfamide requires prospective randomized trials. In sequential phase II trials, increasing the dose of doxorubicin from 50 mg/m$^2$ to 75 mg/m$^2$ in combination with infosfamide 5 gm/m$^2$ appeared to result in improved efficacy.[22,23] Yet, in a randomized trial by the same group, there was no difference in response or survival.[21] In the only randomized trial in which the dose of infosfamide was the major variable, the EORTC reported a response rate of 3% among patients treated with ifosfamide 5 g/m$^2$ by 24-hour infusion, but 17% among patients who received 9 g/m$^2$ given by 4-hour infusion daily for 3 days.[24] The explanation of the low observed response rate observed in the 'standard dose' arm remains a matter of speculation, but may be related to the high proportion of patients with leiomyosarcoma among the study population. This EORTC trial did not employ the extremely high doses of ifosfamide described in other studies, although the results do support the concept of a dose–response relationship.

The strategy generally employed to increase the dose-intensity of infosfamide has been to increase the amount of drug given every three to four weeks. In our study, the strategy was to increase the frequency of ifosfamide administration to every two weeks. In so doing, the total dose administered, expressed in gm/m$^2$/week was as high as any previously reported regimen. Although acute toxicity, including myelosuppression, was not immediately dose-limiting, in the population treated most patients did not receive more than four cycles of therapy. During the treatment the majority of patients were able to accept treatment on schedule. Nonetheless, these patients with their advanced disease and with a limited life-expectancy often stated that continuation of this regimen was inconsistent with their goal of maintaining a good quality of life. Based on current practice at the time this study was planned, the treatment was given entirely on an inpatient basis. This almost certainly was a confounding factor in patient 'burnout'. In addition, the use of erythropoietin might have reduced the fatigue in patients who became anemic during the course of treatment. Today, the regimen we report could be given on an outpatient basis to most individuals. Indeed, after the preliminary report of the present trial,[25] another group also demonstrated the feasibility of repeating cycles of ifosfamide-containing chemotherapy to patients with sarcoma every 2 weeks.[26]

In a phase II trial at Memorial Sloan-Kettering Cancer Center, edatrexate induced responses in 13% of patients with advanced sarcoma.[7] Although the activity of edatrexate in a National Cancer institute of Canada trial was marginal,[27] responses have been seen in an ECOG trial.[8] A high response rate in the latter trial would provide impetus for further exploration of the combination of edatrexate and ifosfamide.

Perhaps the greatest potential for any active drug or regimen lies in adjuvant therapy. Several uncontrolled trials[19,20] and one prospective randomized trial[29] suggest that pre-operative or post-operative anthracycline/ifosfamide combination chemotherapy regimen results in improved survival in patients with operable soft tissue sarcoma. Whereas dose-intense regimens are difficult to apply in patients with advanced disease, experience in other diseases documents that vigorous, repetitive chemotherapy programs can be tolerated by patients for finite periods of time.[30] The present study demonstrates the feasibility of administering a dose-intense schedule of ifosfamide alone or ifosfamide with edatrexate.

### Acknowledgements

This work was supported by NIH Grant CA47179.

### References

1 Antman KH, Ryan L, Elias A, Sherman D, Grier HE. Response to ifosfamide and mesna: 124 previously treated patients with metastatic or unresectable sarcoma. *J Clin Oncol* 1989; 7: 126–31.

2 Bramwell VHC, Mouridsen HT, Santoro A, *et al.* Cyclophosphamide versus ifosfamide: final report of a randomized phase II trial in adult soft tissue sarcomas. *Eur J Cancer Clin Oncol* 1987; 23: 311–21.

3 Patel SR, Vadhan-Raj S, Papadopolous, N, Plager C, Burgess MA, Hays C, *et al.* High-dose ifosfamide in bone and soft tissue sarcomas: results of phase II and pilot studies—dose-response and schedule dependence. *J Clin Oncol* 1997; 15: 2378–84.

4 Antman KS, Griffin JD, Elias A, *et al.* Effect of recombinant human granulocyte-macrophage colony-stimulating factor on chemotherapy-induced myelosuppression. *N Engl J Med* 1988; 319: 593–8.

5 Vadhan-Raj S, Broxmeyer HE, Hittelman WN, Papadopoulos NE, Chawla SP, Fenoglio C, *et al.* Abrogating chemotherapy-induced myelosuppression by recombinant granulocyte-macrophage colony-stimulating factor in patients with sarcoma: protection at the progenitor cell level. *J Clin Oncol* 1992; 10: 1266–77.

6 Elias AD, Eder JP, Shea T, Begg CB, Frei III E, Antman KH. High-dose ifosfamide with mesna uroprotection: a phase I trial. *J Clin Oncol* 1990; 8: 170–8.

7 Casper ES, Christman KI, Schwartz GK, Johnson B, Brennan MF, Bertino JR. Edatrexate in patients with soft tissue sarcoma. Activity in malignant fibrous histiocytoma. *Cancer* 1993; 72: 766–70.

8 Wasserheit C, Blum RH, Ryan L. Phase II trial of edatrexate in adult patients with metastatic soft tissue sarcomas, an ECOG phase II trial. *Proc Am Soc Clin Oncol* 1998; 17: 513a.

9 Li WW, Lin JT, Tong WP, Trippett TM, Brennan MF, Bertino JR. Mechanisms of natural resistance to antifolates in human soft tissue sarcomas. *Cancer Res* 1992; 52: 434–8.

10 Schmid FA, Sirotnak FM, Otter GM, DeGraw JI. Combination chemotherapy with a new folate analog: activity of 10-ethyl-10-deaza-aminopterin compared to methotrexate with 5-fluorouracil and alkylating agents against advanced metastatic disease in murine tumor models. *Cancer Treat Rep* 1987; 71: 727–32.

11 Adult Intergroup Soft-Tisse Sarcoma Committee. Recommendations for clinical protocol development and definition of therapeutic response for soft tissue sarcoma. *Cancer* 1980; 46: 796–800.

12 Antman K, Crowley J, Balcerzak SP, Rivkin SE, Weiss GR, Elias A, *et al.* An Intergroup phase III randomized study of doxorubicin and decarbazine with or without ifosfamide and mesna in advanced soft tissue and bone sarcomas. *J Clin Oncol* 1993; 11: 1276–85.

13 Edmonson JH, Ryan LM, Blum RH, Brooks JS, Shiraki M, Frytak S, *et al.* Randomized comparison of doxorubicin alone versus ifosfamide plus doxorubicin or mytomycin, doxorubicin, and cisplatin against advanced soft tissue sarcomas. *J Clin Oncol* 1993; 11: 1269–75.

14 Santoro A, Tursz T, Mouridsen H, Verweij J, Steward W, Somers R, *et al.* Doxorubicin versus CYVADIC versus doxorubicin plus ifosfamide in first-line treatment of advanced soft tissue sarcomas: a randomized study of the European Organization for Research and Treatment of Cancer Soft Tissue and Bone Sarcoma Group. *J Clin Oncol* 1995; 13: 1537–45.

15 Rosen G, Forscher C, Lowenbraun S, Eilber F, Eckardt J, Holmes C, *et al.* Synovial sarcoma. Uniform response of metastases to high dose ifosfamide. *Cancer* 1994; 73: 2506–11.

16 Buesa J, Lopez-Pousa A, Anton A, Martin J, Garcia del Muro J, Bellmunt J, *et al.* Phase II trial of first-line, high-dose ifosfamide (HD-IF) in advanced soft tissue sarcoma (STS) patients. *Proc Am Soc Clin Oncol* 1997; 16: 498a.

17 Singer JM, Hartley JM, Brennan C, Nicholson PW, Souhami RL. The pharmacokinetics and metabolism of infosfamide during bolus and infusional administration; a randomized cross-over study. *Br J Cancer* 1998; 77: 978–84.

18 Le Cesne A, Antoine E, Spielmann M, Lee Chevalier T, Brian E, Toussaint C, *et al.* High-dose ifosfamide: circumvention of resistance to standard-dose ifosfamide in advanced soft tissue sarcomas. *J Clin Oncol* 1995; 13: 1600–8.

19 Reichardt P, Tilgner J, Hohenberger P, Dorken B. Dose-intensive chemotherapy with ifosfamide, epirubicin, and filgrastim for adult patients with metastatic of locally advanced soft tissue sarcoma: a phase II trial. *J Clin Oncol* 1998; 16: 1438–43.

20 Patel SR, Vadhan-Raj S, Burgess MA, Plager C, Padadoplous N, Jenkins J, Benjamin RS. Results of two consecutive trials of dose-intensive chemotherapy with doxorubicin and ifosfamide in patients with sarcomas. *Am J Clin Oncol* 1998; 21(3): 317–21.

21 Tursz T, Verweij J, Judson I, Crowther D, Le Cesne A, Somers R, Keiser J, *et al.* Is high-dose chemotherapy of interest in advanced soft tissue sarcomas (ASTS)? An EORTC randomized Phase III trial. *Proc Am Soc Clin Oncol* 1996; 15:337.

22 Schutte J, Mouridsen HT, Steward W, *et al.* Ifosfamide plus doxorubicin in previously untreated patients with advanced soft tissue sarcoma. *Eur J Cancer* 1990; 26: 558–61.

23 Steward WP, Verweij J, Somers R, Spooner D, Kerbat P, Clavel M, *et al.* Granulocyte-Macrophage Colony-Stimulating Factor allows safe escalation of dose-intensity of chemotherapy in metastatic adult soft tissue sarcomas: a study of the European Organization for Research and Treatment of Cancer soft Tissue and Bone Sarcoma Group. *J Clin Oncol* 1993; 11: 15–21.

24 Van Oosterom AT, Krzemienlecki K, Nielson OS, Judson I, Mouridsen HT, Svancarova L, *et al.* Randomized phase II study of the EORTC Soft Tissue and Bone Sarcoma (STSBS) group comparing two different ifosfamide (IF) regimens in chemotherapy untreated advanced soft tissue sarcoma (STS) patients (pts). *Proc Am Soc Clin Oncol* 1997; 16: 496a.

25 Christman KL, Casper ES, Schwartz GK. High intensity scheduling of ifosfamide in adult patients with soft tissue sarcoma. *Proc. Am Soc Clin Oncol* 1993; 12: 470.

26 Pronzato P, Losardo P, Pensa F, Tognoni A. High-dose-intensity combination chemotherapy for advanced sarcomas: a pilot study. *Cancer Chemother Pharmacol* 1998; 41: 513–6.

27 Knowling M, Bramwell V, Eisenhauer E, Boos G, Bodurtha A, Quirt I. Phse II trial of 10-EDAM in advanced soft tissue sarcoma. A study of the Canadian Sarcoma Group and the National Cancer Institute of Canada Clinical Trials Group. *Ann Oncol* 1994; 5: 766–8.

28 Spiro IJ, Suit H, Gebhardt M, Springfield D, Mankin H, Jennings C, Rosenberg A, *et al.* Neoadjuvant chemotherapy and radiotherapy for large soft tissue sarcomas. *Proc Am Soc Clin Oncol* 1996; 15: 524.

29 Frustaci S, Gherlinzoni F, De Paoli A, Pignatti G, Zmerly H, Azzarelli A, *et al.* Preliminary results of an adjuvant randomized trial on high risk extremity soft tissue sarcomas (STS). The interim analysis. *Proc Am Soc Clin Oncol* 1997; 16: 496a.

30 Hudis C. Sequential dose-dense adjuvant therapy with doxorubicin, paclitaxel, and cyclophosphamide. *Oncology* 1997; 11: 15–8.
